# Diphtheria

**Published:** 1864-02

**Authors:** 


### DIPHTHERIA.

Dr. J. West Walker presents (*British Med. Journal,* May 16, 1863,) some interesting views relative to this disease which are worthy of consideration. He maintains that the true *nature* of diphtheria must be very different from that hitherto received. "We can no longer," he remarks, "consider it to be an acute specific disease, having uniform general and local symptoms. The leather-like formation, hitherto held to be the diagnostic sign, at once loses its significance, if it have to be viewed only in the light of a complication of nearly every ill that flesh is heir to; manifesting itself, it is true, only at certain seasons, such seasons being noted for the extensive prevalence of zymotic diseases generally." He does not deny "that, during a diphtheritic epidemic, a distinct, and, to a certain extent, new zymotic diseases may possibly exist, to which the name diphtheria may, though rather inaptly, be applied; all I maintain is, that if such a disease do exist, we have no positive symptom by which to recognize it; and that, as far as its general symptoms go, they only represent a condition of bloodpoison analogous to, though possibly increased in severity over, diseases already known—presenting differences of degree more than of kind; and that the so-called local pathognomonic formation associated, as it is found to be, with an endless variety of general symptoms, can no longer be employed as a diagnostic sign."

"If, then," he adds, "a variety of general diseases, alike only in having the common diphtheritic complication, are no longer to be considered as one distinct disease to be called diphtheria, the sooner, for all practical purposes, the name be done away with the better, for it cannot but mislead. It conveys not the slightest notion of the true nature of the affection (or affections;) and it renders utterly nugatory all attempts to reduce either diagnosis, prognosis, the question of contagion, or the method of treatment, to a scientific basis. Far better would it be to employ the word in all and every case generally, no matter what the general symptoms may be, wherein the pathognomonic sign presents itself, only reducing it to the rank of a qualifying adjective. We should then speak of cases as diphtheritic, whatever the general symptoms showed the patient to be at the time laboring under. We should be induced to study more closely such co-existing malady, and not being led away by a name, be more likely to form a correct idea of any particular case.

The *theory* of the nature of diphtheria, to be induced from the foregoing facts and observations, may be briefly stated in the following conclusions, viz:

1.—The characteristic formation is but an external complication, and has no specific relation to any particular state of system.

2.—The general symptoms with which this formation is found to be associated are most various; ranging from the most trifling *malaise* to the most virulent septicæmia, and extending through the whole class of acute specific diseases.

3.—Possibly, during the prevalence of a diphtheritic epidemic, there may be a distinct general disease, altogether different from other known diseases; but we have no positive evidence on the subject.

4.—Diphtheria, in the sense in which the word has hitherto been employed, is to be looked upon not as one disease, but rather as many diseases alike only in being associated with the common characteristic formation.—*American Journal Medical Sciences.*

